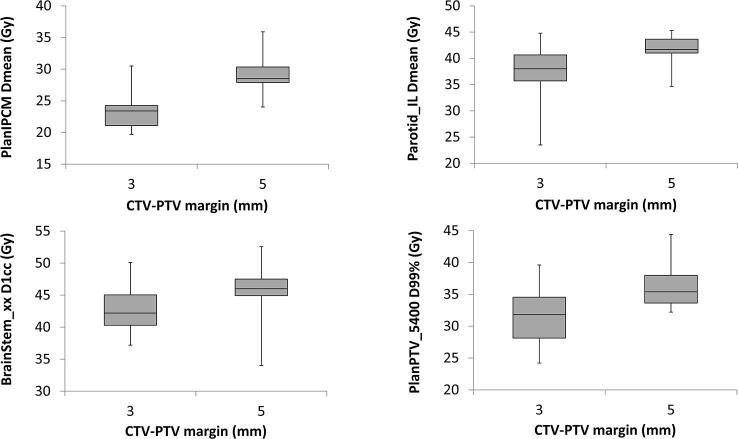# Quality assurance of dysphagia-optimised intensity modulated radiotherapy treatment planning for head and neck cancer

**DOI:** 10.1016/j.phro.2021.10.003

**Published:** 2021-10-26

**Authors:** Justine Tyler, David Bernstein, Matthew Seithel, Keith Rooney, Imran Petkar, Elizabeth Miles, Catharine H Clark, Emma Hall, Chris Nutting

**Affiliations:** a National Radiotherapy Trials Quality Assurance Group, The Royal Marsden NHS Foundation Trust, 203 Fulham Road, London SW3 6JJ, UK; b The Institute of Cancer Research, 123 Old Brompton Road, London SW7 3RP, UK; c Radiotherapy Department, The Royal Marsden NHS Foundation Trust, 203 Fulham Road, London SW3 6JJ, UK; d National Radiotherapy Trials Quality Assurance Group, Mount Vernon Cancer Centre, Northwood, Middlesex HA6 2RN, UK; e Radiotherapy Physics, University College London Hospital NHS Foundation Trust, 5th Floor West, 250 Euston Road, NW1 2PG, UK; f Department of Medical Physics and Biomedical Engineering, University College London, WC1E 6BT, UK; g Metrology for Medical Physics, National Physical Laboratory, Hampton Rd, Teddington, TW11 0PX, UK

**Keywords:** CERR, Computational Environment for Radiotherapy Research, DARS, dysphagia/aspiration related structures, DICOM, Digital Imaging and Communications in Medicine, DO-IMRT, dysphagia optimised intensity modulated radiotherapy, ICR-CTSU, Clinical Trials and Statistics Unit and the Institute of Cancer Research, IPCM, inferior pharyngeal constrictor muscle, NIHR, National Institute for Health Research, PAF, plan assessment form, RTQA, radiotherapy quality assurance, RTTQA, UK’s National Radiotherapy Trials Quality Assurance Group, S-IMRT, standard intensity modulated radiotherapy, SMPCM, superior and middle pharyngeal constrictor muscles, TMG, Trial Management Group, Intensity modulated radiotherapy (IMRT), Volumetric arc therapy (VMAT), Head and neck cancer, Dysphagia, Randomised controlled trial, Quality assurance

## Abstract

This study aimed to assess the impact of the margin applied to the clinical target volume, to create the planning target volume, on plan quality of a novel dysphagia-optimised intensity modulated radiotherapy technique developed within a head and neck cancer multicentre randomised controlled trial. Protocol compliant plans were used for a single benchmark planning case.

Larger margins were associated with higher doses to adjacent organs at risk, particularly the inferior pharyngeal constrictor muscle, but coincided with some improved low dose target coverage. A 3 mm margin is recommended for this technique if local practices allow.

## Highlights

• A novel dysphagia-optimised technique was developed within a clinical trial.
• All participating centres achieved protocol compliant final plans.
• Larger CTV-PTV margins were associated with higher doses to adjacent OAR.
• A 3 mm margin is recommended for dysphagia-optimisation if local practices allow.

## 1 Introduction

Swallowing difficulty is a significant side effect for people who have been treated with radiation for head and neck cancer (HNC) [1], [2]. Dysphagia-optimised intensity modulated radiotherapy (DO-IMRT) has been shown to reduce dysphagia within a multicentre randomised controlled trial (RCT) by reducing dose to pharyngeal constrictor muscles (PCM) [3], [4], [5], [6].

Poor protocol compliance negatively impacts on clinical trial outcomes, compliance can be improved by radiotherapy trial quality assurance (RTQA) [7], [8], [9], [10], [11], [12], [13], [14]. RTQA is particularly important in HNC RCTs where radiotherapy complexity has increased over time. However, an assessment of DO-IMRT plan quality implemented at multiple centres in an RCT has not been previously reported. This study aimed to assess the impact of the margin applied to the clinical target volume to create the planning target volume (CTV-PTV margin) on DO-IMRT plan quality of protocol compliant plans within an RCT.

## 2 Material and methods

A total of 24 centres submitted plans for the DO-IMRT planning benchmark case that were protocol-compliant at final submission as part of the dysphagia/aspiration related structures (DARS) RCT (CRUK/14/014) described in more detail in supplementary material A [3], [4], [15]. Centres were encouraged to treat using volumetric modulated arc therapy (VMAT) since this improved dose distributions in comparison with fixed field IMRT [5], [6]. The range of techniques and treatment planning systems (TPS) are detailed in supplementary material B.

Internationally recommended standardised nomenclature was used summarised in Table A.1 [16]. Two CTVs were provided; CTV_6500 included the primary or nodal gross target volume (GTV) with a 1 cm isotropic margin, CTV_5400 included areas at risk of microscopic disease. The PCMs were provided as two structures; the superior and middle part (SMPCM) and the inferior part (IPCM) [17], [18]. Other provided organs at risk (OAR) included the spinal cord, brainstem and parotids [19]. CTVs were grown by an isotropic margin to create PTVs according to each centre’s protocol. Plan volumes were edited for dose reporting purposes. PlanPTV_6500 was PTV_6500 cropped from body surface receiving 65 Gy in 30 fractions. PlanPTV_5400 was PTV_5400 cropped from body surface and from PTV_6500 receiving 54 Gy (±1 Gy) in 30 fractions. Planning Organ at Risk Volumes (PRVs) were created by applying margins to the spinal cord and brainstem according to each centre’s protocol.

**Fig. 1:**
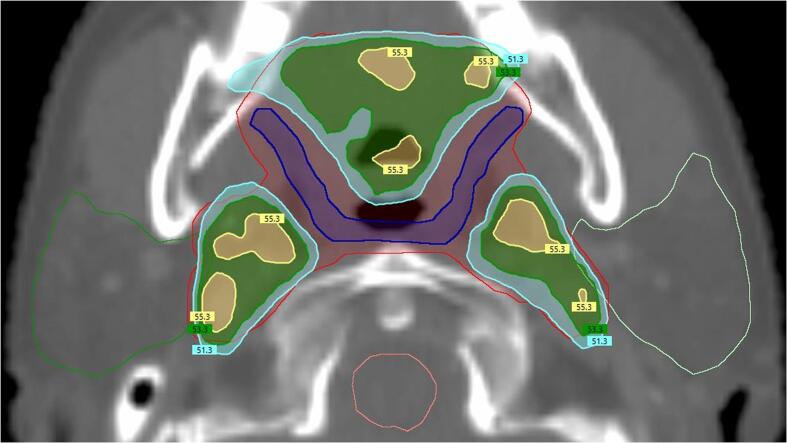
Example DO-IMRT dose distribution showing 95% (51.3 Gy isodose in cyan) coverage of PlanPTV_5400 (red) compromised only in the region of PlanSMPCM (blue) in order to reduce dose whilst maintaining coverage elsewhere. Parotid outlines are displayed in green and brainstem outline in pink. (For interpretation of the references to colour in this figure legend, the reader is referred to the web version of this article.)

The dose was prescribed to the median of the PlanPTVs in accordance with International Commission on Radiation Units & Measurements (ICRU) report 83 [20]. Optimal and mandatory dose-volume constraints are given in the Table A.2 [6], [21]. Specifically, SMPCM cropped from CTV_6500, PlanSPMCM, had a mandatory 50 Gy mean dose constraint for oropharynx cases. Whilst IPCM cropped from CTV_6500, PlanIPCM, had an optimal 20 Gy mean dose constraint for oropharynx cases. PlanPTV_5400 coverage could be compromised to achieve PCM constraints, but not PlanPTV_6500 coverage. Good target dose homogeneity and coverage was required in non-overlap regions (see example in Fig. 1). Planning aims were prioritised in the following order: critical organ constraints (spinal cord and brainstem), PlanPTV_6500 coverage, PCM constraints, PlanPTV_5400 coverage, parotid constraints, other non-specified normal tissue.

A planning benchmark case was chosen to be representative (oropharynx, typical PlanPTV_6500 vol 119 cc). A CT image (2.5 mm slice thickness) with CTVs and OARs outlined was provided. Approval to use patient data was sought. Centres created PTVs, PRVs and plan structures and produced a DO-IMRT plan. Centres were requested to resubmit if they did not meet the mandatory trial requirements or if optimal requirements were not sufficiently met. Knowledge sharing was provided to centres when requested [22].

Plans were assessed using plan assessment forms (PAF), detailing dose-volume constraints, as well as reviewing the full dose distribution using Computational Environment for Radiotherapy Research (CERR) software [23]. The impact of CTV-PTV margins on plan quality from protocol compliant plans, using the PAFs, was quantified. As the dataset was not normally distributed, medians were calculated, and the different margins compared using a Mann-Whitney test (Matlab version R2016b), as the two groups were considered independent due to being from different centres and/or treatment planning systems. The results were considered statistically significant if the p-value was less than 0.05.

## 3 Results

From the 24 centres there were 26 plans in total, as two centres changed their TPS therefore re-planned. Four initial submissions were assessed as acceptable, fourteen initial submissions were resubmitted once and eight initial submissions were resubmitted twice (total of 56 submissions). The reasons for resubmission are detailed in supplementary material C. All the final benchmark planning submissions complied with trial protocol and QA guidelines. The median time taken from first submission to approval was 12 weeks (ranging from 1 to 35 weeks). When the trial opened to recruitment four centres were QA approved and fourteen centres had started pre-trial QA.

Centres used locally determined CTV-PTV margins, the magnitude of which varied amongst different centres to reflect the uncertainties present in their treatment process, including contouring accuracy, immobilisation techniques, on-treatment imaging and machine uncertainty. A 3 mm margin was used in 11 plans, 4 mm in 4 plans, and 5 mm in 11 plans. Plans using 4 mm margins were not compared due to insufficient numbers.

The results comparing dose-volume metrics between plans using a 3 mm margin to those using a 5 mm margin are summarised in Table 1. Statistically significant reductions were found for the 3 mm plans in comparison to the 5 mm plans for; PlanIPCM mean dose (median reduction 5.1 Gy, p < 0.01); ipsilateral parotid mean dose (median reduction 3.7 Gy, p = 0.03); brainstem PRV D_1 cc_ (median reduction 3.8 Gy, p = 0.04); and, PlanPTV_5400 D_99%_ (median reduction 3.6 Gy, p = 0.02). Supplementary figure includes box plots to show dose-volume metrics where there were significant differences. The optimal PlanIPCM constraint (<20 Gy) was not achieved by most plans, being achieved by only three plans, all using a 3 mm margin.

**Table 1:** Dose-volume metrics for plans with 3 mm CTV-PTV margins vs. 5 mm CTV-PTV margins. For the PRVs, xx is the margin in mm (e.g. 03 for 3 mm). Example metric: D_99__%_ is the minimum dose delivered to 99% of the structure. *Optimal constraints did not have to be achieved.

Structure	Dose-volume metric	Constraint	3 mm CTV-PTV margin Median (IQR)	5 mm CTV-PTV margin Median (IQR)	Mann-Whitney *U* test p-value
PlanSMPCM	D_mean_ (Gy)	<50.0	49.4 (48.8–49.6)	49.1 (48.8–49.6)	0.82
PlanIPCM	D_mean_ (Gy)	<20.0*	23.4 (21.1–24.3)	28.5 (27.9–30.4)	<**0.01**
Parotid_CL	D_mean_ (Gy)	<24.0*	26.7 (24.1–28.7)	29.9 (27.7–30.9)	0.08
Parotid_IL	D_mean_ (Gy)	<24.0*	38.0 (35.7–40.7)	41.7 (41.0–43.7)	**0.03**
SpinalCord	D_max_ (Gy)	<48.0	42.8 (39.1–44.3)	43.0 (41.2–45.4)	0.51
	D_1 cc_ (Gy)	<46.0	38.6 (36.2–40.9)	39.4 (38.6–42.9)	0.41
SpinalCord_xx	D_max_ (Gy)	<48.0	41.2 (38.8–43.1)	44.3 (42.5–46.0)	0.05
BrainStem	D_max_ (Gy)	<55.0	44.2 (43.1–47.0)	47.6 (44.8–48.6)	0.26
	D_1 cc_ (Gy)	<54.0	38.6 (37.7–42.1)	42.3 (40.7–43.8)	0.19
BrainStem_xx	D_max_ (Gy)	<55.0	42.2 (40.3–45.1)	46.0 (44.9–47.5)	**0.04**
PlanPTV_6500	D_99%_ (Gy)	>58.5	62.2 (61.4–62.4)	61.9 (61.2–62.1)	0.16
	D_98%_ (Gy)	>61.8*	62.6 (62.3–62.9)	62.4 (61.8–62.6)	0.16
	D_95%_ (Gy)	>61.8	63.3 (62.8–63.5)	63.0 (62.6–63.3)	0.32
	D_50%_ (Gy)	=65.0	65.0 (65.0–65.1)	65.0 (65.0–65.0)	0.67
	D_mean_ (Gy)		65.0 (64.9–65.0)	64.9 (64.8–65.0)	0.19
	D_5%_ (Gy)	<68.3	66.4 (66.1–66.6)	66.3 (66.1–66.7)	0.95
	D_2%_ (Gy)	<69.6	66.7 (66.3–67.0)	66.6 (66.5–67.2)	0.87
PlanPTV_5400	D_99%_ (Gy)		31.8 (28.1–34.6)	35.4 (33.6–38.0)	**0.02**
	D_98%_ (Gy)		40.3 (36.2–41.8)	42.2 (40.2–43.6)	0.09
	D_95%_ (Gy)		47.3 (45.8–49.0)	48.7 (48.0–49.3)	0.18
	D_50%_ (Gy)	<55.0, >53.0	54.3 (54.0–54.5)	54.1 (54.1–54.3)	0.41
	D_mean_ (Gy)		53.8 (53.6–54.3)	53.8 (53.7–54.1)	0.67
	D_5%_ (Gy)		60.0 (59.2–60.9)	59.2 (58.6–59.6)	0.16
	D_2%_ (Gy)		61.8 (61.2–63.0)	61.6 (60.9–62.1)	0.69

Values in bold are statistically significant (<0.05).

## 4 Discussion

Larger CTV-PTV margins were associated with higher doses to some OARs, as expected for PlanIPCM and the ipsilateral parotid due to the greater PTV overlap and their constraints not being mandatory. The constraints for these structures were more closely achieved by 3 mm margin plans compared to 5 mm margin plans. Brainstem PRV doses were higher for 5 mm margin plans however the mandatory constraint was achieved by all plans. The Brainstem PRV was closer to PTVs using a 5 mm margin, meaning that it was not possible to reduce the dose as much as when using a 3 mm margin. PlanSMPCM mean dose was not significantly different as the mandatory constraint was achieved by all plans and was not reduced further than necessary due to overlap with PTV_5400. Previous studies have shown that reduction in dose to OARs due to reduced margins can result in reduced toxicity whilst maintaining sufficient target coverage [24], [25], [26].

The reduction in PlanPTV_5400 D_99%_ for 3 mm margin plans relative to 5 mm margin plans, could be interpreted as larger margins providing better PlanPTV_5400 dose coverage at the expense of PlanIPCM dose. The optimal PlanIPCM constraint was either achieved, or almost achieved, by all centres using 3 mm margin but not by those using 5 mm margin. This may be partly explained by the 5 mm margin PTVs having a smaller proportion of their volume overlapping with PlanIPCM, making it easier to increase PlanPTV_5400 coverage. It is also possible that centres using a 3 mm margin may be more inclined to try to achieve the PlanIPCM constraint, accepting compromise in PlanPTV_5400 coverage as it should be prioritised in this way. However, centres using a 5 mm margin may not optimise to the same degree as the optimal PlanIPCM constraint is not achievable, therefore it would be preferable to accept less compromise in PlanPTV_5400 coverage.

These findings do not account for the possible effect of other factors, including delivery technique, TPS and the planner subjectivity. These factors were excluded from the analysis due to the limited size of the dataset. However, the large variation in final dose distributions, particularly when considering the range of some OAR doses, suggests there are other significant factors impacting the dose distributions.

Most centres used a VMAT delivery technique as recommended, therefore comparison to fixed field IMRT and TomoTherapy® was not possible. TPSs vary in terms of their optimisation and calculation algorithms, degree of user interaction, time taken to optimise and machine delivery limitations. Centres were not advised on specifically how to optimise their plans unless advice was sought or if plans required significant improvement. There was not enough data to quantify the effect of TPS on plan quality, however this has been investigated previously [27].

Although the guidelines were carefully written to minimise misinterpretation and ensure consistency, there was still scope for the user to apply their own judgement in terms of what was perceived to be the best plan quality and what was prioritised by clinicians. Unlike mandatory constraints, how acceptable it is to deviate from an optimal constraint could be considered to be open to interpretation, leading to more variation. The use of structures and constraints not specified in the trial guidelines, may have also contributed to the variation between centres by potentially impacting the specified dose constraints.

Although dose-volume constraints for PTVs were provided, these did not describe the full 3D dose distribution and do not guarantee an acceptable dose distribution. In the case of PlanPTV_5400, it was not possible to define reasonable dose-volume constraints due to PCM overlap, leading to potentially significant variation. To avoid this impacting the trial outcomes, variation was minimised by requesting resubmission if there was poor conformality, coverage or homogeneity. Automated planning may assist with reducing subjectivity in optimisation of plans and provide a more objective gold standard to compare to [28].

This study only applies to one representative case, it does not consider the effect of different cases and their impact in terms of size of outlined structures, their proximity to each other, and the difficulty in achieving constraints. It has been shown that reduced size of target volumes leads to improved dysphagia outcomes [29]. This study also does not account for the effect of treating cases in a typical timeframe, with the associated time pressures in outlining and planning, or individual case considerations. Prospective individual case reviews of plans are used to prevent these pressures impacting plan quality. It would be useful to determine whether successful completion of pre-trial QA leads to good quality radiotherapy during the trial and whether case reviews are necessary to ensure this. RTQA is essential to achieving good compliance to trial protocols and impacts outcomes [7], [8], [9], [10], [11], [12], [13], [14]. However, resources are limited and the timeframe required to complete prospective reviews is short, therefore it needs to be justified.

This study benefits from the co-operation of multiple centres following a standardised DO-IMRT technique defined and controlled within an RCT. All centres successfully completed the pre-trial QA following a well-established process [14]. Centres completing pre-trial QA represented a wide range of techniques and TPSs in use. Although it has not been possible to investigate all variables in detail, the size of the CTV-PTV margin has been identified as a key component which can affect plan quality. There were no consistent differences in IGRT strategy or equipment between centres using 3 mm or 5 mm margins.

All centres completing the benchmark plan were able to achieve acceptable DO-IMRT plans complying with the trial. More than one submission was usually required due to unfamiliarity with the DO-IMRT technique. Larger margins were associated with higher doses to some OARs. However, centres using a 5 mm margin may be achieving better PlanPTV_5400 coverage at the expense of PlanIPCM dose, therefore accounting for some of the differences. A 3 mm margin is recommended for effective DO-IMRT if local practices allow.

## Declaration of Competing Interest

The authors declare that they have no known competing financial interests or personal relationships that could have appeared to influence the work reported in this paper.

## Figures and Tables

**Supplementary Fig. 1:**