# Supplementary material for: Quality assurance of dysphagia-optimised intensity modulated radiotherapy treatment planning for head and neck cancer
Source: Phys Imaging Radiat Oncol. 2021 Oct 26;20:46–50. doi: 10.1016/j.phro.2021.10.003 (PMC8560997; doi:10.1016/j.phro.2021.10.003)
Supplement: Supplementary Data 1 [file mmc1.docx]

**Supplementary figure**

Box plots to show dose-volume metrics where there was a statistically significant difference between plans using a 3 mm CTV-PTV margin compared to a 5 mm CTV-PTV margin.

**Supplementary material A: Summary of the dysphagia/aspiration related structures (DARS) trial and Radiotherapy Quality Assurance (RTQA)**

The DARS trial (CRUK/14/014) was a phase III randomised multicentre study of DO-IMRT versus S-IMRT in patients receiving HNC treatment. Its objective was to determine if a reduction in the radiation dose delivered to the PCM using DO-IMRT improved swallowing function compared to S-IMRT, without increasing recurrence rates. The trial randomised 112 patients between 2016 and 2018.

The trial required RTQA for the implementation of DO-IMRT at participating sites, provided by the national RTTQA Group. RTQA followed a national credentialing programme, which included completion of a process questionnaire, pre-trial benchmarking outlining and planning (DO-IMRT) QA case and prospective review of at least the first two patients recruited at each centre. This helped centres implement the protocol and QA guidelines and was designed to ensure the quality and consistency of treatments under the trial.

The pre-trial QA process for each participating centre included: submission of a process questionnaire containing specific information on the radiotherapy process relevant to the trial, assessment of clinician’s consistency and protocol compliance in target and organ delineation by completing an oropharynx outlining benchmark case, assessment of the centre’s DO-IMRT plan quality for a provided oropharynx planning benchmark case, and a dosimetry audit.

Table A.1: Standardised nomenclature for volumes in the DARS trial

| **Name** | **Description** |
| --- | --- |
| Body | Body outline |
| CTV_6500 | CTV to receive a dose of 65 Gy |
| PTV_6500 | Uncropped PTV to receive a dose of 65 Gy |
| PlanPTV_6500 | PTV cropped from body surface if required for reporting purposes, to receive a dose of 65 Gy |
| CTV_5400 | CTV to receive a dose of 54 Gy. Multiple CTV_5400 combined for reporting doses. |
| PTV_5400 | Uncropped PTV to receive a dose of 54 Gy |
| PlanPTV_5400 | PTV cropped from body surface and from PTV_6500 for reporting purposes, to receive a dose of 54 Gy |
| SpinalCord | Spinal cord |
| SpinalCord_xx | Spinal cord PRV, where xx is the margin in mm (e.g. 03 for 3 mm) |
| BrainStem | Brainstem |
| BrainStem_xx | Brainstem PRV, where xx is the margin in mm (e.g. 03 for 3 mm) |
| Parotid_IL | Ipsilateral Parotid |
| Parotid_CL | Contralateral Parotid |
| SMPCM | Superior & Middle Pharyngeal Constrictor Muscle (contoured as one structure) |
| PlanSMPCM | SMPCM cropped from CTV_6500 |
| IPCM | Inferior Pharyngeal Constrictor Muscle |
| PlanIPCM | IPCM cropped from CTV_6500 |

Table A.2: Dose-volume constraints in the DARS trial

| **Structure** | **Constraint** | **Mandatory** | **Optimal** |
| --- | --- | --- | --- |
| PlanPTV_6500 | D_99%_ | > 90% |  |
|  | D_98%_ |  | > 95% |
|  | D_95%_ | > 95% |  |
|  | D_50%_ | = 100% |  |
|  | D_5%_ | < 105% |  |
|  | D_2%_ | < 107% |  |
| PlanPTV_5400 (S-IMRT) | D_99%_ | > 90% |  |
|  | D_98%_ |  | > 95% |
|  | D_95%_ | > 95% |  |
|  | D_50%_ | = 100% (±1 Gy) |  |
|  | D_5%_ |  | As low as possible |
|  | D_2%_ |  | As low as possible |
| PlanPTV_5400 (DO-IMRT) | D_99%_ |  | As high as possible |
|  | D_98%_ |  | As high as possible |
|  | D_95%_ |  | As high as possible |
|  | D_50%_ | = 100% (±1 Gy) |  |
|  | D_5%_ |  | As low as possible |
|  | D_2%_ |  | As low as possible |
| Spinal cord | Maximum dose | < 48 Gy |  |
|  | D_1 cc_ | < 46 Gy |  |
| Spinal cord PRV | D_1 cc_ | < 48 Gy |  |
| Brainstem | Maximum dose | < 55 Gy |  |
|  | D_1 cc_ | < 54 Gy |  |
| Brainstem PRV | D_1 cc_ | < 55 Gy |  |
| Contralateral Parotid | Mean dose | as low as possible | < 24 Gy |
| Ipsilateral Parotid | Mean dose | as low as possible | < 24 Gy |
| PlanSMPCM (DO-IMRT oropharynx) | Mean dose | < 50 Gy |  |
| PlanIPCM (DO-IMRT oropharynx) | Mean dose |  | < 20 Gy |
| PlanSMPCM (DO-IMRT hypopharynx) | Mean dose |  | < 40 Gy |
| PlanIPCM (DO-IMRT hypopharynx) | Mean dose | < 50 Gy |  |

**Supplementary material B: Distribution of techniques and treatment planning systems**

Table B.1 details the number of centres using different planning techniques. The majority of centres used arc therapy as it was recommended in order to best achieve homogeneous coverage of the PTVs and satisfy the dose-volume constraints to the OARs. Fixed field IMRT was acceptable, however it was anticipated from a local planning study that it would be more challenging to achieve optimal dose distributions and may reduce the likelihood of achieving the optimal constraints.

Table B.1: Distribution of techniques across participating centres

| **Technique** | **Number of centres** |
| --- | --- |
| VMAT | 22 |
| TomoTherapy® | 1 |
| fixed field IMRT | 1 |

Table B.2 details the distribution of treatment planning systems for submitted benchmark plans. From the 24 centres there were 26 plans in total, as two centres changed their TPS therefore repeated the benchmark plan.

Table B.2: Distribution of treatment planning systems for submitted benchmark plans

| **Treatment planning system** | **Number of submissions** |
| --- | --- |
| Varian Eclipse^TM^ | 13 |
| RaySearch RayStation | 4 |
| Oncentra® Masterplan | 4 |
| Philips Pinnacle | 3 |
| Elekta Monaco® | 1 |
| Accuray Tomotherapy®. | 1 |

**Supplementary material C: Reasons for DO-IMRT benchmark plan resubmission**

Table C.1 summarises the reasons for plan resubmission at the first submission and those that were remaining at the second submission (all were acceptable by the third submission). Most of the variations were not detectable by PAF assessment alone. Review of the full 3D dose distribution was required to ensure compliance to the trial protocol, particularly for the PlanPTV_5400 where some compromise in coverage was accepted.

The most common reason for resubmission was due to insufficient PlanPTV_6500 95% isodose coverage. This was usually in the region where dose to PlanSMPCM was reduced where it overlaps in the CTV-PTV margin.

PCM constraints were prioritised over PlanPTV_5400 coverage however it was important to ensure coverage elsewhere was acceptable. Some cases prioritised parotid constraints over PlanPTV_5400 coverage which was not acceptable within the trial.

Small hot areas were accepted if they were not considered to be clinically significant by the reviewing clinician. However if these areas were deemed to be too large and/or located in normal tissue, plan resubmissions were requested and more homogeneous and conformal dose distributions were achieved.

Although the PlanIPCM mean dose constraint was not mandatory and most centres did not achieve it, centres were expected to optimise this constraint as much as possible. The most effective way to ensure this was to compare to the mean dose achieved by other centres using the same CTV-PTV margin which successfully completed the benchmark plan.

The PlanSMPCM constraint was mandatory although some centres did not achieve it initially.

If centres did not follow the instructions for creating plan structures correctly, these had to be resubmitted to ensure dose reporting was accurate and dose-volume constraints were achieved.

Although the parotid mean dose constraints were optimal and most centres did not achieve them, it was considered unacceptable if they were significantly higher compared to other centres using the same CTV-PTV margin which had successfully completed the benchmark plan.

Table C.1: Reasons for plan resubmission at first and second submission

|  | **Submissions** | |
| --- | --- | --- |
| **Variation** | **1st** | **2nd** |
| PlanPTV_6500 95% isodose coverage insufficient | 13 | 2 |
| PlanPTV_5400 95% isodose coverage insufficient away from PCMs | 9 | 3 |
| PAF incomplete or incorrect | 9 | 1 |
| Significant hot areas > 107% of 54 Gy prescription away from PlanPTV_6500 | 8 | 2 |
| PlanIPCM mean dose could be reduced compared to centres using same CTV-PTV margin | 6 | 1 |
| Trial nomenclature not used | 5 | 1 |
| PlanSMPCM mandatory mean dose constraint not achieved | 4 | 2 |
| Plan structures incorrectly created | 4 | 0 |
| Parotid mean dose could be reduced compared to centres using same CTV-PTV margin | 2 | 1 |
